# Impact of a Structured Training Program on Medical Student Confidence and Behavior During Their First Radial Arterial Puncture: Comparative Study

**DOI:** 10.2196/78086

**Published:** 2026-02-18

**Authors:** Camille Rolland-Debord, Lucien Juret, Mathilde Simon, Abdallah El Mouhajer, Cécile Londner, Capucine Morélot-Panzini, Cécile Chenivesse, Thomas Similowski

**Affiliations:** 1 Service de Pneumologie, CHU Clermont-Ferrand, Université Clermont Auvergne, 58 Rue Montalembert, Clermont-Ferrand, 63000, France, 33 4 73 75 16 53; 2 Service de Pneumologie, Département R3S, F-75013, Groupe Hospitalier Universitaire, Assistance Publique–Hôpitaux de Paris, Sorbonne Université, Hôpital Pitié-Salpêtrière, Paris, France; 3 Service des Pathologies du Sommeil, Hôpital Pitié-Salpêtrière, Département R3S, F-75013, Groupe Hospitalier Universitaire, Assistance Publique–Hôpitaux, Sorbonne Université, Paris, France; 4 Neurophysiologie Respiratoire Expérimentale et Clinique, F-75005, INSERM UMRS1158, Sorbonne Université, Paris, France; 5 Service de Pneumologie et Immuno-Allergologie, Centre Hospitalier Universitaire de Lille, Lille, France; 6 Département R3S, F-75013,Hôpital Pitié-Salpêtrière, Groupe Hospitalier Universitaire, Assistance Publique–Hôpitaux, Sorbonne Université, Paris, France

**Keywords:** arterial puncture, medical education, simulation-based training, procedural confidence, medical students, local anesthesia, clinical skills, competency-based learning

## Abstract

**Background:**

Radial artery puncture is a common clinical procedure essential for assessing gas exchange but is frequently perceived as stressful by inexperienced operators, who fear causing pain to their patients. Despite its practical relevance, formal training in this procedure is inconsistently integrated into medical curricula. This study evaluated whether a structured training program—combining theoretical instruction, simulation-based practice, and debriefing—could influence students’ procedural confidence and decision-making and patient experience during their first clinical arterial puncture.

**Objective:**

This study aimed to determine whether structured simulation-based training influences medical students’ anxiety, confidence, and technical performance and patient experience during their first arterial puncture.

**Methods:**

Third-year medical students who had never performed an arterial puncture were assigned to 1 of 2 groups: a structured training group (group 1) or a control group receiving informal or no specific training (group 2). After performing their first arterial puncture under supervision, students completed a questionnaire assessing apprehension, satisfaction, and confidence. The decision to use local anesthesia, puncture success, and patient-rated pain and apprehension were also recorded. A total of 67 students participated (group 1: n=24, 35.8%; group 2: n=43, 64.2%), with 61 patients included. Statistical comparisons were performed using the Fisher exact and nonparametric Mann-Whitney *U* tests (α=.05).

**Results:**

Self-reported apprehension and confidence were similar between groups. However, group 1 students were significantly less likely to use local anesthesia compared to group 2 students (7/20, 35% vs 28/36, 77.8%, respectively; *P*=.003), suggesting greater procedural confidence. First-attempt success rates were comparable (group 1: 3/13, 23.1%; group 2: 14/29, 48.3%; *P*=.18). Median patient-reported pain scores were numerically but not statistically significantly lower when anesthesia was used (2.1, IQR 1.2‐4.0 vs 4.8, IQR 2.1‐6.4; *P*=.08).

**Conclusions:**

Structured training influenced students’ behavior during their first arterial puncture, reducing reliance on anesthesia despite similar levels of self-reported apprehension. Although confidence ratings did not differ, behavioral indicators suggested improved self-efficacy and readiness for clinical performance. These findings support the behavioral impact of structured procedural education and call for future research using validated assessment tools and long-term follow-up.

## Introduction

Performing a radial arterial puncture to assess gas exchange can be challenging for medical and nursing students due to the risk of causing significant pain at a small puncture site densely packed with nociceptive C-fiber–containing structures, including the artery itself and nearby tissues such as the periosteum [1,2]. Several studies have explored strategies to improve patient tolerance. Topical anesthetics have shown limited effectiveness [3,4], and cryoanalgesia, though promising, is seldom used [5]. Local infiltration anesthesia offers the greatest pain reduction but is considered impractical and is infrequently applied [6-8]. Moreover, reducing needle gauge does not alleviate pain and may prolong the procedure [9]. Patout et al [9] demonstrated that preprocedural anxiety in patients significantly correlates with increased pain perception during radial arterial puncture. Importantly, patient anxiety is likely to be heightened by the anxiety of the operator, whose fear of causing discomfort can lead to anticipatory stress. This, in turn, may further complicate the procedure, creating a vicious circle that amplifies the risk of pain.

Structured training programs have been shown to reduce anxiety and build confidence in nursing [10,11] and medical students [12-14] performing clinical procedures. In the particular case of radial arterial puncture, Collins et al [13] implemented a 5-step curriculum for respiratory care students combining theory, simulation, and peer assessment, which resulted in high interrater performance scores. Similarly, simulation-based training has been shown to enhance confidence and technical skills in core procedures among medical students, including those involving invasive techniques [15,16].

In this context, we conducted a national survey of arterial puncture teaching practices in French pneumology departments and found that only a minority used structured programs to train medical students (Multimedia Appendix 1). At our own institution, a systematic observation of unsupervised arterial puncture attempts by third-year medical students on a simulator revealed a high frequency of procedural errors (Multimedia Appendix 1).

Building on these observations and the available literature, we designed a structured training program combining theoretical instruction, simulation-based practice, and supervised hands-on experience. The aim of this study was to evaluate whether adding a structured theoretical and simulation-based training session influenced medical students’ procedural confidence, preprocedural anxiety, and first-attempt performance during radial arterial puncture compared with usual heterogeneous bedside teaching. We hypothesized that structured preparation would be associated with increased confidence and reduced anxiety, as well as a possible improvement in performance.

## Methods

### Ethical Considerations

This study was approved by the institutional review board of the National Institute of Health and Medical Research (reference IRB00003888). All data were collected anonymously, and no personal identifiers were recorded at any stage. Informed consent was obtained from all participants prior to study participation. Participation was voluntary, and students could decline or withdraw at any time without any academic or clinical consequences. No financial or other compensation was provided for participation.

### Recruitment Procedure

This study was conducted in hospitals affiliated with the Faculty of Health, Sorbonne University, one of the medical schools in the Paris area.

All third-year medical students (General Medical Sciences Diploma) completing a 3-month clinical rotation in pulmonology were eligible for inclusion if they performed their first arterial puncture on a patient and had not previously received simulation-based training for this procedure. Each eligible student was invited to complete the online questionnaire within 24 hours of performing the procedure (Multimedia Appendix 1). At the time of rotation assignment, students selecting a pulmonology placement were verbally informed that they would be invited to complete a short online questionnaire after performing their first arterial puncture. This information was reiterated at the beginning and end of the rotation and again during the end-of-semester pulmonology examination. At the start of each rotation, senior physicians in the participating departments were informed of the study via email and provided with a link to the questionnaire for distribution to their students. Data were collected using an online questionnaire specifically developed for this study and hosted on the SurveyMonkey platform (SurveyMonkey Inc; Multimedia Appendix 1). The initial draft was developed by the study investigators and reviewed for face and content validity by a panel of 5 academic pulmonologists and medical educators from Sorbonne University. The instrument was pilot-tested among medical students before implementation.

### Student Groups

#### Overview

Students were assigned to groups based solely on their clinical placement independently of the study investigators. Both groups followed the usual teaching of their respective departments. These practices were heterogeneous and could involve bedside instruction by a nurse, a more senior medical student, a resident, or an attending physician. In some cases, students were verbally instructed before entering the patient’s room or performed the procedure after having observed it on another patient. No intervention was made to modify these existing teaching practices. Regarding the clinical procedure itself, the use of topical anesthesia was left up to the students in accordance with local departmental policies. The students were divided as follows:

- Group 1—students rotating in the respiratory medicine department of Pitié-Salpêtrière Hospital, Sorbonne University, who received the usual teaching plus an additional structured training program before their first arterial puncture
- Group 2—students rotating in other respiratory medicine departments of Sorbonne University who received usual teaching only

For sensitivity analysis, group 2 was then subdivided into students who had received bedside demonstration or guidance before their first arterial puncture (group 2+) and those who had not (group 2−). This subdivision allowed us to capture the full spectrum of existing informal learning situations within the “usual practice” group.

#### Structured Training Program (Group 1)

##### Overview

Students in group 1 participated in a structured theoretical and practical training session that had been routinely implemented in the department in previous years. The session was delivered by a single instructor and organized into 2 groups of approximately 7 students each. The total duration varied slightly between sessions but was at least 1 hour. The session began with a structured discussion of the indications and techniques for arterial puncture, followed by a briefing outlining the simulation objectives, procedural steps to be reproduced, and key safety principles.

##### Theoretical Component

A dedicated slide deck (Multimedia Appendix 1) was developed based on the *New England Journal of Medicine* resource on radial artery puncture [17] incorporating annotated screenshots from the procedural video. The content was reviewed for accuracy and pedagogical coherence by 5 academic pulmonologists. Each student received a printed copy of the material at the end of the session and was granted permanent online access.

##### Practical Component

**Figure 1.**
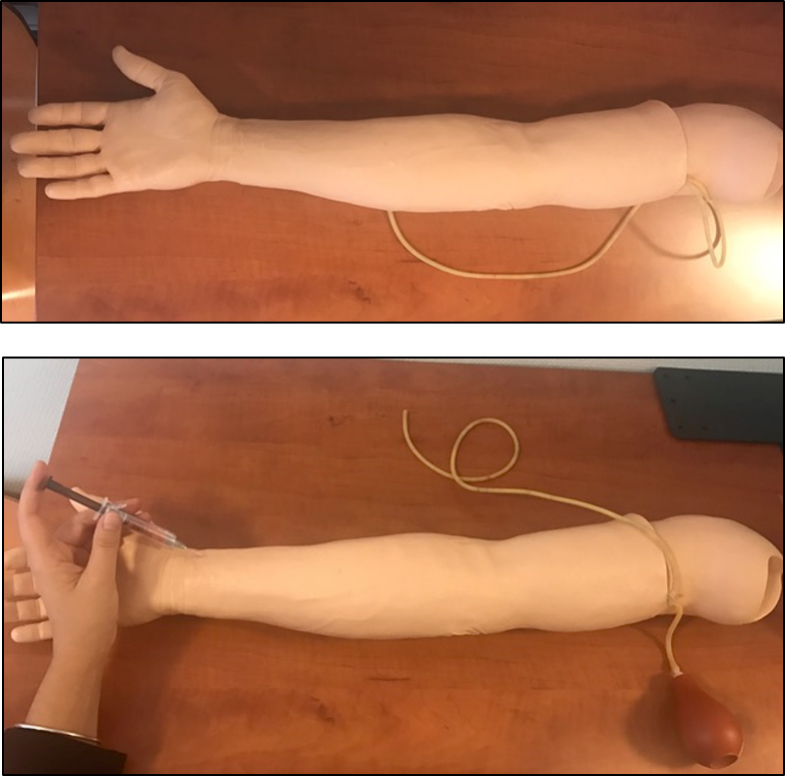
Procedural arm simulator with a pseudopulsatile artery and circulating red liquid (Laerdal Medical).

The practical portion of the session consisted of individual hands-on training using an artificial arm simulator (Laerdal Medical; Figure 1), which allowed for palpation of a simulated pulse and visualization of arterial blood return. Each student repeated the procedure until they achieved self-reported confidence.

Procedural steps were practiced systematically, including patient communication, site preparation, needle positioning and insertion, troubleshooting in case of failure, and needle disposal. Students were encouraged to observe and discuss each other’s attempts to reinforce learning through peer observation.

Peer-to-peer exercises were also incorporated, including palpation of the radial artery groove, performance of the Allen test, patient communication, and patient positioning and preparation.

A standardized communication script (Multimedia Appendix 1) written and reviewed by the same 5 academic pulmonologists was introduced and read aloud by the instructor and then practiced in pairs by students. Each participant received a printed copy for review before performing the procedure in a clinical setting. The script included a standard introduction, an explanation of the procedure, information regarding expected discomfort, and clarification about the absence of unambiguously validated analgesic strategies.

##### Structured Debriefing

Each session concluded with a structured debriefing conducted according to the Haute Autorité de Santé simulation guidelines. Students were first invited to express their emotions and initial impressions (reaction); then analyze their actions and underlying reasoning (analysis); and, finally, summarize key learning points and objectives for improvement (synthesis). Incorrect decisions or technical errors were explored to identify their causes and were addressed through guided feedback, theoretical reinforcement, and targeted procedural adjustments. Throughout the session, the instructor fostered psychological safety; encouraged self-assessment; used open-ended questioning; and provided constructive, nonjudgmental feedback.

At the conclusion of the session, students were reminded that they would later be asked to complete an online questionnaire following their first arterial puncture on a patient.

### Usual Teaching Only (Group 2)

Students in group 2 received the usual teaching routinely provided in the departments to which they had been assigned. Teaching practices varied and could include bedside instruction and/or verbal explanations given before entering the patient’s room. Instruction could be provided by a nurse, a senior medical student, a resident, or an attending physician. In some cases, students performed the procedure after first observing it on another patient. No intervention was introduced to modify these usual teaching practices.

### Evaluation Criteria

Evaluation criteria included several dimensions of student and patient experience. For students, the following variables were assessed: global anticipatory apprehension, apprehension about causing pain, apprehension about procedural failure, satisfaction with the procedure, apprehension about repeating the procedure, and confidence in performing it again. The decision to use topical anesthesia was also recorded, as well as the success rate of the arterial puncture. For patients, perceptions of the procedure were evaluated through 2 measures: anticipatory apprehension and pain experienced during the puncture. Both students and patients rated their perceptions using a 10-cm visual analogue scale (VAS) ranging from 0 (“none at all”) to 10 (“maximum imaginable”).

### Statistical Analysis

Statistical analyses were performed using Prism (GraphPad Software). Categorical variables were reported as counts and percentages, whereas continuous variables were presented as means and SDs or medians and IQRs depending on their distribution. Given the limited sample size and the ordinal nature of several variables, nonparametric statistics were used. The primary analysis compared group 1 and group 2. Group comparisons were conducted as follows: (1) categorical variables were compared using the Fisher exact test, and (2) continuous variables were compared using the Mann-Whitney *U* test. A sensitivity analysis was performed to compare group 1 with the group 2 subgroups: group 2+ (students who received prior training) and group 2− (students who did not receive prior training). All tests were 2 sided, with the type I error rate set at α=.05.

## Results

### Participants

**Figure 2.**
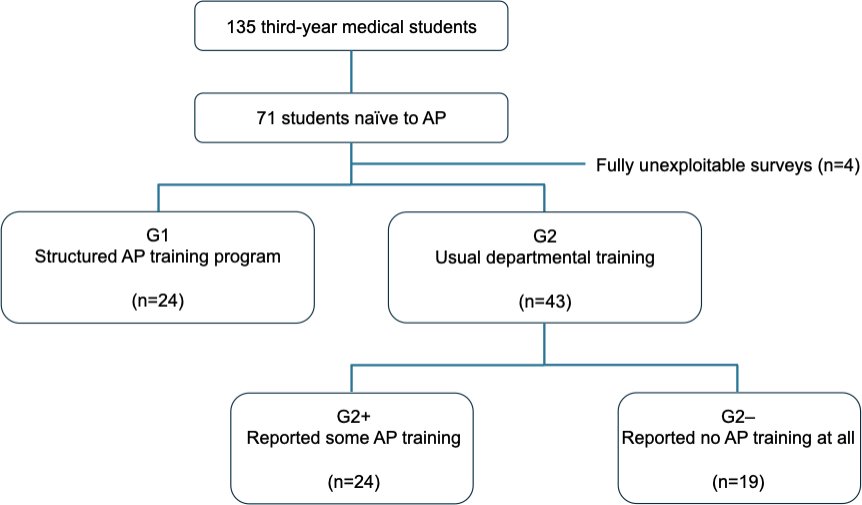
Flowchart of the study. AP: arterial puncture; G1: students assigned to the combined theoretical and practical structured AP training program; G2: students assigned to usual teaching; G2+: subgroup of G2 consisting of students who reported receiving some prior AP training; G2−: subgroup of G2 consisting of students who reported receiving no prior AP training at all.

During the study period, 135 third-year medical students were assigned to the participating departments. Of these 135 students, 71 (52.6%) were entirely naive to arterial puncture and performed their first procedure during their rotation (Figure 2). The cohort included 78.9% (56/71) women and 21.1% (15/71) men, with a median age of 21 (IQR 20‐21) years; 90.1% (64/71) were right-handed. A total of 83.1% (59/71) had previously observed an arterial puncture performed by a nurse or physician. All participants began completing the survey immediately after the procedure, but 21.1% (15/71) submitted incomplete responses. These included 16.7% (4/24) of the students from group 1, a total of 16.3% (7/43) from group 2, and 5.6% (4/71) whose group affiliation could not be determined. These cases were excluded from analysis, yielding a final sample of 67 students. Group 1 included 35.8% (24/67) of the students. Among the 43 students in group 2, a total of 19 (44.2%) reported having received no prior training (group 2−), and 24 (55.8%) reported some form of training (group 2+), which typically consisted of theoretical explanations and observation of procedures performed by nurses, senior students, or physicians.

### Attendants

In total, 92.9% (52/56) of the students were accompanied during their first arterial puncture (19/19, 100% in the group 2− subgroup). Attendants were nurses in 52.2% (35/67) of cases, medical trainees in 31.3% (21/67) of cases (including fourth- to sixth-year medical students and residents more advanced in training than the study participants, who were all in their third year), and senior physicians in the remaining 16.4% (11/67) of cases.

### Medical Students’ Perceptions

**Figure 3.**
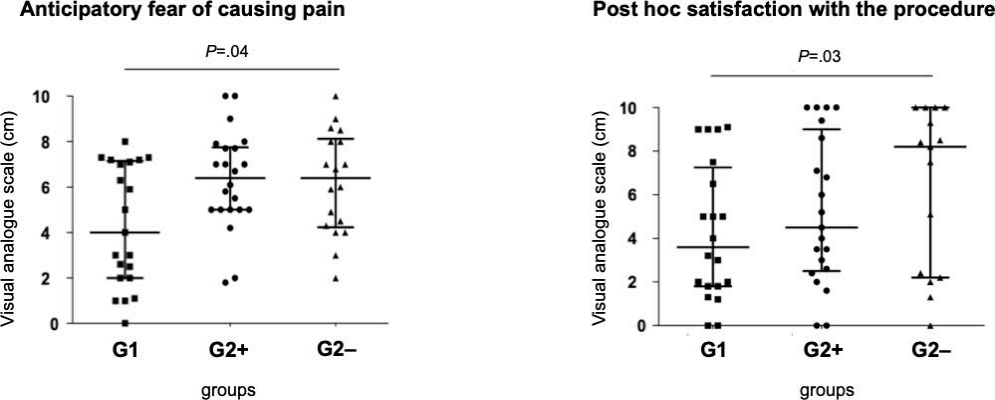
Sensitivity analysis. G1: students assigned to the combined theoretical and practical structured arterial puncture training program; G2+: subgroup of group 2 consisting of students who reported receiving some prior arterial puncture training; G2−: subgroup of group 2 consisting of students who reported receiving no prior arterial puncture training at all.

No statistically significant differences were observed between group 1 and group 2 in terms of global anticipatory apprehension, apprehension about causing pain, apprehension about procedural failure, satisfaction with the procedure, apprehension about repeating the procedure, or confidence in repeating the procedure (Table 1). However, anticipatory apprehension about causing pain was significantly lower in group 1 compared to group 2− (median 4.0, IQR 2.0‐7.0 vs median 6.4, IQR 4.2‐8.5; *P*=.04; Table 2 and Figure 3). Conversely, postprocedure satisfaction was significantly higher in group 2− than in group 1 (median 8.2, IQR 2.3‐10.0 vs median 3.6, IQR 1.8‐7.2; *P*=.03; Table 2 and Figure 3).

**Table 1.** Students’ perception of the procedure: group 1 vs group 2.^a^

	Group 1, median (IQR)	Group 2, median (IQR)	*P* value
Global anticipatory apprehension	4.1 (2.2-5.0)	4.0 (2.0-5.6)	.67
Apprehension about causing pain	5.0 (2.3-7.2)	5.0 (3.1-6.9)	.60
Apprehension about procedure failure	4.3 (2.3-6.2)	4.4 (2.0-6.9)	.84
Satisfaction with the procedure	3.6 (1.8-7.3)	5.6 (2.4-9.4)	.09
Apprehension about repeating the procedure	1.9 (0-4.6)	2.3 (0.3-3.9)	.87
Confidence in repeating the procedure	7.0 (5.1-7.8)	6.0 (4.4-8.0)	.80

a Group 1: students assigned to the combined theoretical and practical structured training program (data from 20/24, 83.3% of the students; incomplete survey forms excluded). Group 2: other students (data from 43/46, 93.5% of the students; incomplete survey forms excluded). Comparisons between the groups were performed using the Mann-Whitney *U* test.

**Table 2.** Students’ perception of the procedure: group 1 vs group 2–.^a^

	Group 1, median (IQR)	Group 2–, median (IQR)	*P* value
Global anticipatory apprehension	4.2 (1.0-6.1)	4.7 (1.7-6.2)	.44
Apprehension about causing pain	4.0 (2.0-7.0)	6.4 (4.2-8.5)	.04
Apprehension about procedure failure	5.0 (3.0-7.0)	5.2 (3.0-7.2)	.56
Satisfaction with the procedure	3.6 (1.8-7.2)	8.2 (2.3-10.0)	.03
Apprehension about repeating the procedure	1.8 (0-4.6)	2.5 (1.0-3.8)	.58
Confidence in repeating the procedure	7.0 (5.0-7.7)	6.0 (5.0-9.2)	.87

a Group 1: students assigned to the combined theoretical and practical structured training program (n=24). Group 2–: subgroup of group 2 consisting of students who reported receiving no prior training (n=19). Comparisons between the groups were performed using the Mann-Whitney *U* test.

### Use of Topical Anesthesia

Overall, 62.5% (35/56) of the students elected to use anesthesia before performing the arterial puncture. Among them, 45.7% (16/35) used an EMLA 5% patch, 51.4% (18/35) used EMLA 5% cream, and 2.9% (1/35) used subcutaneous lidocaine. Anesthesia use was significantly less frequent in group 1 compared to group 2 (7/20, 35% vs 28/36, 77.8%; *P*=.003). The use of anesthesia did not lead to statistically significant differences in students’ perceptions or patients’ reported experiences, although procedural pain and self-reported confidence showed nonsignificant trends (Table 3).

**Table 3.** Patients’ and students’ experience based on the use of preprocedure anesthesia. Comparisons between groups were performed using the Mann-Whitney *U* test.

	Without prior anesthesia, median (IQR)	With prior anesthesia, median (IQR)	*P* value
Patients			
Anticipatory apprehension	4 (2-6.2)	3 (1.5-5.7)	.50
Procedural pain	4.8 (2.1-6.4)	2.1 (1.2‐4)	.08
Students			
Global anticipatory apprehension	3.8 (1.8-5.8)	4.1 (2-5.1)	.89
Apprehension about causing pain	5 (2-7.3)	5 (3.4-6.9)	.57
Apprehension about procedure failure	4.3 (2-7.2)	4.4 (2-6.2)	.82
Satisfaction with the procedure	4 (2-9)	5.1 (2‐8.8)	.61
Apprehension about repeating the procedure	1.9 (0-5)	2.4 (0-4.1)	.93
Confidence in repeating the procedure	7.1 (5.3-8.6)	5.4 (4.3-7.9)	.07

### Arterial Puncture Success

The first-attempt success rate was 23.1% (3/13) in group 1 and 48.3% (14/29) in group 2 (*P*=.18). Within group 2, students with no prior training (group 2−) achieved a notably higher first-attempt success rate than those with some prior training (group 2+): 71.4% (10/14) vs 28.6% (4/14), respectively (*P*=.06). The overall success rate within the first 3 attempts was 65% (13/20) in group 1 and 80.6% (29/36) in group 2 (*P*=.20). The median number of attempts required for success was significantly higher in group 1 than in group 2 (median 1, IQR 0.5‐2 vs median 1, IQR 0‐1; *P*=.02). The number of procedures requiring supervisor intervention after 3 failed attempts was identical in both groups (7/20, 35% and 7/36, 19.4%).

### Patient Description and Experiences

Arterial punctures were performed by students on 61 patients (median age 65, IQR 57‐76 years; n=38, 62.3% women and n=23, 37.7% men). The number of patients (n=61) was lower than the number of participating students because some students performed their first arterial puncture on the same patient for logistical reasons. A total of 26.2% (n=16) of the patients were receiving oral analgesics, primarily paracetamol. Most patients (n=41, 67.2%) had previously undergone multiple radial arterial punctures.

Overall, anticipatory apprehension was rated at a median of 3.5 (IQR 2.0‐6.0) on the VAS, with no statistically significant difference between patients who did not receive anesthesia and those who did (median 4.0, IQR 2.0‐6.2 vs median 3.0, IQR 1.5‐5.7; *P*=.50). Overall procedural pain was rated at a median of 2.7 (IQR 1.2‐5.4). Pain scores were higher in the group without anesthesia than in the group with anesthesia (median 4.8, IQR 2.1‐6.4 vs median 2.1, IQR 1.2‐4.0), although the difference did not reach statistical significance (*P*=.08).

## Discussion

### Principal Findings

In a context where the training provided to French medical students for arterial puncture appears highly variable and generally brief—typically lasting less than 30 minutes—and where unsupervised students without specific training demonstrate frequent procedural errors (Multimedia Appendix 1), our results show that a structured training program can meaningfully influence behavior. Specifically, students who completed the structured program (group 1) were significantly less likely to choose local anesthesia before the procedure compared to those who received only informal or no training (group 2; 7/20, 35% vs 28/36, 77.8%; *P*=.003) despite having similar levels of self-reported anticipatory apprehension. This occurred even though the rate of successful punctures did not differ significantly between groups. From the patients’ perspective, no statistically significant differences were found in anticipatory apprehension or procedural pain based on anesthesia use. Nonetheless, the difference in pain ratings exceeded the minimal clinically important difference on a VAS—typically estimated at 13 mm (1.3 cm) [18]—suggesting a clinically meaningful effect even in the absence of statistical significance. These findings are consistent with those of prior studies that report limited impact of topical anesthetics and highlight the role of operator anxiety in shaping patient experience [4,5,9].

Recent reviews in respiratory care education highlight that simulation—whether of low or high fidelity—enhances clinical reasoning, adherence to safety standards, and learner engagement [14]. To our knowledge, this study is among the first to examine the impact of structured training on medical students performing their first arterial puncture. While no direct parallels exist in this population, similar findings have been reported in other health care student groups. For example, Collins et al [13] implemented a 5-step curriculum for respiratory care students combining theory, simulation, and peer assessment, which resulted in high interrater performance scores. In nursing education, structured programs using artificial arm simulators have improved procedural success rates and reduced errors [11], and a competency-based model allowed nurses to reach proficiency levels typically associated with physicians [19]. Given these precedents—and the findings from our preliminary observations (Multimedia Appendix 1)—our study focused not on procedural skill acquisition but on the impact of structured training on students’ apprehension and confidence. In other domains, simulation-based training and structured debriefing have been shown to reduce anxiety and enhance self-confidence. For instance, Schmidt et al [20] demonstrated reduced anxiety in students presenting clinical cases after receiving video-based feedback, and Toy et al [21] reported improved confidence and competence following simulation-based training in core invasive procedures. Similar outcomes have been reported with academic detailing, an approach that typically refers to individualized educational outreach for practicing clinicians [22] that can influence clinical decision-making and has also been shown to improve confidence, competence, and critical thinking [23]. In our study, students who completed the structured training program (group 1) reported levels of apprehension and confidence similar to those of students with informal or no training (group 2) both before and after the procedure. While these results might initially appear less favorable, group 1 students were significantly less likely to use local anesthesia (7/20, 35% vs 28/36, 77.8%; *P*=.003), revealing a notable divergence between self-reported perception and actual behavior. This suggests that the training influenced confidence in ways not fully captured by subjective ratings. Choosing to perform a first arterial puncture without anesthesia may reflect a stronger sense of preparedness despite lingering concerns about causing pain. Supporting this interpretation, group 1 students showed significantly less apprehension about causing pain than students with no prior training (group 2−). Group 1 students also reported lower postprocedure satisfaction than group 2− students, which may reflect more realistic expectations shaped by the training and a narrower gap between anticipation and experience. The lower use of local anesthesia by group 1 students may also be due to the educational content of the program, which did mention the limited effectiveness of topical anesthetics for arterial puncture according to the literature. However, group 1 students were not advised against their use and were taught how to administer them. This leads us to believe that increased self-confidence did play a role in the observed difference, possibly in addition to or in synergy with adherence to the course content.

This study has several limitations. First, the structured training was not delivered by certified simulation instructors as recommended by the French National Authority for Health. This may have affected its quality—particularly the debriefing, widely regarded as the most critical element of simulation-based learning. Second, the educational interventions received by students in both groups were inherently heterogeneous. Teaching practices varied across hospital sites, ranging from bedside instruction by a nurse, senior medical student, resident, or attending physician to situations in which students performed the procedure with minimal prior observation. This design intentionally reflected the authentic diversity of clinical training conditions rather than an artificially standardized environment to assess the added value of a structured program under real-world conditions. Such heterogeneity may have introduced uncontrolled differences in baseline exposure. Future studies could address this by comparing structured training with a standardized “usual practice” curriculum to more precisely isolate the effects of the structured training. Third, we relied on nonvalidated VASs to assess students’ apprehension and confidence. While these were practical and easy to administer, their psychometric properties are uncertain, limiting the reliability and generalizability of the findings. Future studies should consider using validated instruments for anxiety and procedural self-efficacy, as well as integrating frameworks such as entrustable professional activities to better capture learners’ readiness for unsupervised clinical practice. Fourth, contextual and patient-related factors could not be controlled across hospital sites. Differences in patient characteristics or case complexity may have influenced the technical difficulty of arterial puncture and contributed to variations in first-attempt success rates. Additional limitations include the potential demotivating effect of a lengthy questionnaire, which may have contributed to incomplete responses and the absence of data on the time interval between training and the procedure. Although students had continuous access to theoretical materials, we cannot confirm that they reviewed them prior to performing the puncture, which may have attenuated the effects of the training.

### Conclusions

Structured training for arterial puncture influences students’ behavior in meaningful ways even when self-reported confidence and apprehension appear unchanged. The reduced reliance on local anesthesia among trained students may reflect greater readiness to take responsibility for a potentially painful procedure. These findings support the value of formalized training and highlight the need for validated tools and long-term evaluation to fully capture its educational and clinical impact. Although exploratory and limited by methodological constraints, this study provides initial evidence that such pedagogical frameworks could be meaningfully integrated into undergraduate medical curricula in pulmonology and related disciplines.

## Supplementary material

10.2196/78086 Multimedia Appendix 1 Survey, teaching slide, and script.

## Abbreviations

VAS: visual analogue scale
