# Supplementary material for: Impact of a Structured Training Program on Medical Student Confidence and Behavior During Their First Radial Arterial Puncture: Comparative Study
Source: JMIR Med Educ. 2026 Feb 18;12:e78086. doi: 10.2196/78086 (PMC12916088; doi:10.2196/78086)
Supplement: Multimedia Appendix 1 [file mededu-v12-e78086-s001.docx]

**Electronic supplement to** *"A structured training program for medical students performing their first arterial puncture: impact on apprehension and confidence",* ROLLAND-DEBORD et al.

## Electronic supplement 1 (ES1): survey of teaching practices in French pneumology departments.

This supplement describes a survey assessing the training provided to French medical students in performing arterial punctures for arterial blood gas analysis within university hospital pneumology departments. The survey (see below) was distributed to 37 department heads via the national teaching association for respiratory medicine (*Collège des Enseignants de Pneumologie*).

Twenty-two departments responded, reporting a median of 15 students per rotation [interquartile range: 9–20]. Of these, one department offered only theoretical instruction, 11 provided only practical training, and 8 included both theoretical and practical components. Two departments reported having no structured training program.

Among the 19 departments offering practical training, 6 (32%) used artificial arms for simulation, while the remaining 13 relied on supervised bedside practice. Regardless of the training modality, the estimated duration was less than 30 minutes in all cases. Training was delivered by a senior physician in 75% of departments and by residents in the remaining 25%.

## Survey questionnaire:

| **ORIGINAL FRENCH VERSION** Questionnaire en ligne destiné aux responsables de services de pneumologie universitaires en France  1. Une formation théorique est-elle dispensée aux étudiants en médecine en stage dans votre service avant la réalisation de ponctions artérielles ? : \|__\| Oui - \|__\| Non  2. Une formation pratique est-elle dispensée aux étudiants en médecine en stage dans votre service avant la réalisation de ponction artérielle ? : \|__\| Oui - \|__\| Non  Si oui, quel en est le support :  \|__\| au lit du malade  \|__\| via l’utilisation d’un mannequin/bras artificiel  \|__\| entraînement sur personnel médical/paramédical  3. Cette formation est réalisée par : \|__\| médecin senior – \|__\| interne – \|__\| externe - \|__\| infirmier – \|__\| élève infirmier - \|__\| cadre de santé  4. Cette formation dure : \|__\|moins de 30 minutes - \|__\| entre 30 et 60 minutes – \|__\| plus de 60 minutes  5. Cette formation est : \|__\|individuelle - \|__\| collective  6. Combien d’étudiants avez-vous en moyenne par trimestre ? | **ENGLISH TRANSLATION** Online questionnaire for the heads of university hospitals respiratory medicine department in France  1. Is a theoretical training provided to medical students during their internship in your department before they perform an arterial puncture? : \|__\| Yes - \|__\| No  2. Is a practical training provided to medical students during their internship in your department before they perform an arterial puncture ?? : \|__\| Yes - \|__\| No  If yes, what is the format:  \|__\| bedside training  \|__\| using a mannequin/artificial arm  \|__\| training on medical/paramedical staff  3. This training is provided by: \|__\| senior physician – \|__\| resident – \|__\| intern - \|__\| nurse – \|__\| nursing student - \|__\| health manager  4. The duration of this training is: \|__\| less than 30 minutes - \|__\| between 30 and 60 minutes – \|__\| more than 60 minutes  5. This training is: \|__\| individual - \|__\| collective  6. How many students do you have on average per quarter? |
| --- | --- |
|  |  |

## Electronical supplement 2 (ES2): observational survey of errors by untrained students.

This survey was conducted in the respiratory medicine department of Pitié-Salpêtrière Hospital, a 1,600-bed tertiary university hospital in Paris, France. It evaluated the unsupervised arterial puncture practices of third-year medical students using a simulation model. The students had only theoretical knowledge of radial arterial puncture, with no prior hands-on experience on patients or simulators, although some had previously observed the procedure.

Each student was asked to perform a radial arterial puncture on a specially designed artificial arm (Laerdal Medical, Stavanger, Norway), under the observation of a senior physician who recorded procedural errors using a pre-established checklist based on clinical practice recommendations. Thirty-one students participated.

The main findings are summarised in Figure ES1. Before the procedure, 55% of students failed to explain the procedure to the simulated patient, 61% did not assess arterial patency (e.g., via Allen’s test), and 45% made errors in glove selection or use. Pre-procedural disinfection was correctly performed in 90% of cases.

During the procedure, 26% of students incorrectly positioned the patient’s arm in extension, 32% misaligned the needle with respect to the arm’s axis, 26% failed to orient the needle correctly toward the artery, and 38% did not use a bevel-up needle position. After the procedure, site compression and safe disposal of the needle and syringe were performed correctly in 90% of cases.

## Checklist derived from care practice recommendations used to evaluate students' errors during supervised radial arterial puncture on a simulation artificial arm.

| **ORIGINAL FRENCH VERSION** Questionnaire à remplir par l'observateur  1. Genre étudiant :  \|__\| Homme \|__\| Femme  2. Âge lors de la ponction artérielle en années :  I__I __I  3. Main dominante :  \|__\| droite \|__\| gauche  4. Premier gaz du sang :  \|__\| Oui \|__\| Non  5. Information du patient avant la ponction artérielle radiale :  \|__\| Oui \|__\| Non  6. Vérification avant la ponction artérielle radiale :  \|__\| rien – \|__\| perméabilité de l'artère cubitale -  \|__\| anticoagulants / coagulopathie - \|__\| autre  7. Mise de gants :  \|__\| aucun – \|__\| stériles – \|__\| non stériles  8. Désinfection réalisée :  \|__\| aucune – \|__\| bétadine alcoolique – \|__\| chlorhexidine –  \|__\| autre  9. Position du poignet du patient :  \|__\| extension – \|__\| position neutre – \|__\| flexion – \|__\| autre  10. Position du biseau :  \|__\| vers le haut – \|__\| vers le bas – \|__\| sur le côté  11. Direction aiguille :  \|__\| vers l’épaule – \|__\| vers le poignet  12. Inclinaison de l’aiguille par rapport à l’axe du bras :  \|__\| moins de 30° - \|__\| 30 à 45 ° - \|__\| 46 à 90 °  13. Compression du point de ponction après la ponction :  \|__\| oui – \|__\| non  14. Traitement de l’aiguille après ponction :  \|__\| maîtrisé – \|__\| non maîtrisé  15. Traitement de la seringue après ponction :  \|__\| maîtrisé – \|__\| non maîtrisé | **ENGLISH TRANSLATION** Observer's checklist  1. Student's gender:  \|__\| Male \|__\| Female  2. Age at the time of arterial puncture (in years):  I__I __I  3. Dominant hand:  \|__\| Right \|__\| Left  4. First blood gas:  \|__\| Yes \|__\| No  5. Patient informed before radial arterial puncture:  \|__\| Yes \|__\| No  6. Checks before radial arterial puncture:  \|__\| None – \|__\| Ulnar artery patency -  \|__\| Anticoagulants/coagulopathy - \|__\| Other  7. Glove usage:  \|__\| None – \|__\| Sterile – \|__\| Non-sterile  8. Disinfection performed:  \|__\| None – \|__\| Alcoholic betadine –  \|__\| Chlorhexidine – \|__\| Other  9. Patient's wrist position:  \|__\| Extension – \|__\| Neutral position – \|__\| Flexion – \|__\| Other  10. Bevel position:  \|__\| Upward – \|__\| Downward – \|__\| Sideways  11. Needle direction:  \|__\| Toward the shoulder – \|__\| Toward the wrist  12. Needle angle relative to the arm's axis:  \|__\| Less than 30° - \|__\| 30 to 45° - \|__\| 46 to 90°  13. Compression of the puncture site after puncture:  \|__\| Yes – \|__\| No  14. Needle handling post-puncture:  \|__\| Mastered – \|__\| Not mastered  15. Syringe handling post-puncture:  \|__\| Mastered – \|__\| Not mastered |
| --- | --- |

## Electronical supplement 3 (ES3): online survey performed within 24 hours of performing their first radial arterial puncture on a patient.

Full version of the questionnaire developed for this study, including both the student and patient sections. Items assess anticipatory apprehension, procedural confidence, satisfaction, pain perception, and use of local anesthesia. Each variable is measured using 10-cm visual analog scales (VAS) or short categorical items. The questionnaire was developed by the study investigators and reviewed for face and content validity by a panel of five academic pulmonologists and medical educators from Sorbonne Université.

| **ORIGINAL FRENCH VERSION**  **Questionnaire en ligne destiné aux étudiants participant au programme de formation**  Vos initiales 1ère lettre du nom /1ère lettre du prénom :  Nom I__I - Prénom I__I  Genre :  \|__\| Homme \|__\| Femme  Quel âge avez-vous, en années :  I__I __I  Votre main dominante :  \|__\| droite \|__\| gauche  S’agit-il bien de votre premier gaz du sang :  \|__\| Oui \|__\| Non  **A. Vos connaissances avant la réalisation de ponction artérielle** **radiale** **1.** Avant votre première ponction artérielle, aviez-vous déjà vu une ponction artérielle chez un patient :  \|__\| Oui \|__\| Non Si oui, des explications sur la réalisation du geste vous avaient-elles été fournies ?  \|__\| Oui \|__\| Non  **2.** Avez-vous reçu une formation lors de ce stage avant la réalisation de la ponction artérielle ?  \|__\| Oui \|__\| Non Si oui : **2a.** Cette formation était :  \|__\| théorique – \|__\| pratique  **2b.** Cette formation était réalisée par :  \|__\| médecin senior – \|__\| interne – \|__\| externe - \|__\| infirmier – \|__\| élève infirmier - \|__\| cadre de santé **2c**. Cette formation était :  \|__\| très utile - \|__\| utile – \|__\| peu utile - \|__\| inutile  **2d**. Cette formation a duré :  \|__\| moins de 30 minutes - \|__\| entre 30 et 60 minutes –  \|__\| plus de 60 minutes **2e**. Cette formation était :  \|__\| individuelle - \|__\| collective **2f.** Si vous avez reçu une formation pratique, quel était le support \|__\| au lit du malade  \|__\| via l’utilisation d’un mannequin/bras artificiel  \|__\| entraînement sur personnel médical/paramédical  **3.** Que faut-il vérifier avant une ponction artérielle radiale ?  \|__\| rien – \|__\| perméabilité de l'artère cubitale - \|__\| anticoagulants / coagulopathie - \|__\| ne sait pas  **4.** Quels gants doit-on porter ?  \|__\| aucun – \|__\| stériles – \|__\| non stériles – \|__\| ne sait pas  **5.** Quelle désinfection doit être réalisée ?  \|__\| aucune – \|__\| bétadine alcoolique –  \|__\| chlorhexidine – \|__\| ne sait pas  **6.** Quelle doit être la position du poignet du patient ?  \|__\| pas de préférence – \|__\| extension – \|__\| position neutre –  \|__\| flexion – \|__\| ne sait pas  **7.** Quelle doit être la position du biseau ?  \|__\| vers le haut – \|__\| vers le bas – \|__\| sur le côté – \|__\| ne sait pas  **8.** De quel côté l’aiguille doit être dirigée ?  \|__\| vers l’épaule – \|__\| vers le poignet – \|__\| ne sait pas  **9.** Quelle doit être l'angle de l’aiguille par rapport à l’axe du bras :  \|__\| moins de 30° - \|__\| 30 à 45 ° - \|__\| 46 à 90 ° - \|__\| ne sait pas  **10.** Doit-on comprimer le point de ponction après la ponction ?  \|__\| oui – \|__\| non – \|__\| ne sait pas  **B. Vos ressentis sur cette ponction artérielle radiale avant sa réalisation ?** (échelle de 0 à 10; 0 : pas de peur, 10 : maximale imaginable)  Peur globale :  I__I __I  Peur de provoquer une douleur au patient :  I__I __I  Peur de rater le geste :  I__I __I   **C. Caractéristiques de votre patient** **Age** en années  I__I __I  **Sexe**  \|__\| Homme \|__\| Femme  **Poids** (kg)  I__I __I __I  **Taille** (cm)  I__I __I __I  **Antécédent cardio-vasculaire - artériopathie**  I__I oui I__I non  **Tabac :**  I__I sevré I__I actif I__I jamais  **Traitement en cours par des antalgiques**  I__I oui I__I non  Si oui, lesquels ...........................................................................................  **D. Vécu de la ponction artérielle radiale par votre patient :**  Expériences antérieures de ponction artérielle radiale  I__I 0 – I__I 1 – I__I 2 – I__I 3 - I__I plus de 3  Douleur liée à la dernière ponction artérielle  (de 0 à 10) : I__I __I  Malaises causés par des gestes techniques faits antérieurement : I__I oui - I__I non Si oui :  I__I ponction veineuse –  I__I ponction artérielle -  I__I autre  À combien votre patient cote sa peur de votre ponction artérielle ? (de 0 à 10) : I__I __I  À combien votre patient côte sa douleur de votre ponction ?  (de 0 à 10) : I__I __I  A quel moment la douleur a été la plus forte :  I__I insertion de l’aiguille  I__I pendant ponction  I__I retrait de aiguille  I__I après ponction  I__I lors de répétition de la ponction  **E. Caractéristiques de votre ponction artérielle radiale :**  Lieu de réalisation de votre ponction artérielle?  \|__\| EFR, \|__\| Hôpital de jour, \|__\| Hospitalisation traditionnelle, \|__\| Réhabilitation, \|__\| Consultation  Accompagnateur lors de votre ponction artérielle?  \|__\| Oui \|__\| Non  Si oui :  I__I médecin senior  I__I interne  I__I externe  I__I infirmier  I__I élève infirmier  Explication donnée au patient avant ponction artérielle :  \|__\| Oui \|__\| Non  Réalisation anesthésie préalable :  \|__\| Oui \|__\| Non  Si oui, quelle méthode ?  \|__\| patch Emla - \|__\| crème Emla - \|__\| lidocaine sous cutanée  Si oui, combien de temps avant :  \|__\| 0 à 30min - \|__\| 30 à 60 min - \|__\| plus de 60 min  Qualité du pouls radial :  I__I imperceptible, I__I faible, I__I normal, I__I visible à œil nu  Poignet ponctionné :  \|__\| droit \|__\| gauche  Diamètre aiguille :  \|__\| 25 G : orange – \|__\| 23 G : bleue – \|__\| autre  Nombre de tentatives avant le succès de ponction artérielle :  \|__\| 0 - I__I 1 – I__I 2 – I__I 3  Échec après 3 tentatives :  \|__\| Oui \|__\| Non  Si oui, causes :  \|__\| hématome \|__\| prélèvement insuffisant, \|__\| artère non ponctionnable \|__\| artère non trouvée, \|__\| intolérance du patient  Si, oui quelle a été la conduite après l’échec ?  \|__\| ponction faite sur autre poignet  \|__\| faite avec une autre aiguille  \|__\| ponction faite par un autre opérateur  \|__\| ponction faite sur un autre site (fémoral, huméral, capillaire) \|__\| ponction non réalisée  **E. Vos ressentis personnels sur cette ponction artérielle radiale pendant sa réalisation**  Peur globale (de 0 à 10) : I__I __I  Peur de provoquer une douleur au patient (de 0 à 10) : I__I __I  Peur de rater le geste (de 0 à 10) : I__I __I   **F.** **Vos ressentis sur cette ponction artérielle radiale après sa réalisation**  Satisfaction de votre geste (de 0 à 10): I__I __I  Peur de refaire le geste (de 0 à 10) : I__I __I  Confiance en soi de refaire le geste (de 0 à 10) : I__I __I | **ENGLISH TRANSLATION**  **Online questionnaire for students participating in the training program**  Your initials (1st letter of last name/1st letter of first name):  Last name I__I - First name I__I  Gender:  \|__\| Male \|__\| Female  How old are you (in years):  I__I __I  Your dominant hand:  \|__\| Right \|__\| Left  Is this your first arterial puncture for blood gas analysis:  \|__\| Yes \|__\| No  **A. Your knowledge before performing radial arterial puncture**  **1.** Before your first arterial puncture, had you ever observed an arterial puncture in a patient?:  \|__\| Yes \|__\| No If yes, were explanations about the procedure provided to you? \|__\| Yes \|__\| No  **2.** Did you receive training during this internship before performing an arterial puncture?  \|__\| Yes \|__\| No If yes: **2a.** The training was:  \|__\| theoretical – \|__\| practical  **2b.** The training was provided by:  \|__\| senior physician – \|__\| resident – \|__\| intern - \|__\| nurse – \|__\| nursing student - \|__\| health manager **2c**. The training was:  \|__\| very useful - \|__\| useful – \|__\| somewhat useful - \|__\| not useful **2d**. The training lasted:  \|__\| less than 30 minutes - \|__\| between 30 and 60 minutes –  \|__\| more than 60 minutes **1e**. The training was:  \|__\| individual - \|__\| collective **1f**. If you received practical training, what was the method?:  \|__\| bedside  \|__\| using a mannequin/artificial arm  \|__\| training on medical staff  **3.** What should be checked before a radial arterial puncture?  \|__\| None – \|__\| Ulnar artery patency - \|__\| Anticoagulants/coagulopathy - \|__\| Don’t know  **4.** Which gloves should be used?  \|__\| None – \|__\| Sterile – \|__\| Non-sterile – \|__\| Don’t know  **5.** Which disinfection should be performed?  \|__\| None – \|__\| Alcoholic betadine – \|__\| Chlorhexidine –  \|__\| Don’t know  **6.** What should be the patient’s wrist position?  \|__\| No preference – \|__\| Extension – \|__\| Neutral –  \|__\| Flexion – \|__\| Don’t know  **7.** What should be the bevel position?  \|__\| Upward – \|__\| Downward – \|__\| Sideways – \|__\| Don’t know  **8.** In which direction should the needle be aimed?  \|__\| Shoulder – \|__\| Wrist – \|__\| Don’t know  9. What should be the needle’s position relative to the arm’s axis?  \|__\| Less than 30° - \|__\| 30 to 45° - \|__\| 46 to 90° - \|__\| Don’t know  **10.** Should the puncture site be compressed after the puncture?  \|__\| Yes – \|__\| No – \|__\| Don’t know  **B. Your feelings about this radial arterial puncture before performing it?** (0-10 scale; 0, no fear; 10: worst imaginable fear)    Overall fear level:  I__I __I  Fear of causing pain to the patient:  I__I __I  Fear of failing the procedure:  I__I __I  **C. Characteristics of your patient Age** (in years)  I__I __I  **Sex**  \|__\| Male \|__\| Female  **Weight** (kg)  I__I __I __I  **Height** (cm)  I__I __I __I  **History of cardiovascular disease - Arteriopathy**  I__I Yes I__I No  **Smoking**  I__I Former smoker I__I Active smoker I__I Never smoked  **Current treatment with painkillers**  I__I Yes I__I No  If yes, which ones ...........................................................................................  **D. Experience of radial arterial puncture by your patient:**  Previous experiences of radial arterial puncture by your patient: I__I 0 – I__I 1 – I__I 2 – I__I 3 - I__I More than 3  Pain related to the last radial arterial puncture:  (0-10 scale) I__I __I    Previous discomfort caused by technical procedures:  I__I Yes I__I No If yes:  I__I Venipuncture  I__I Arterial puncture  I__I Other  How does your patient rate their fear of your arterial puncture?: (0-10 scale) I__I __I  How does your patient rate their pain of your arterial puncture?: (0-10 scale) I__I __I  When was the pain strongest?  I__I Needle insertion  I__I During puncture  I__I Needle removal  I__I After puncture  I__I During repeated puncture  **D. Characteristics of your arterial puncture** Where was the puncture performed?  \|__\| PFT lab \|__\| Day hospital, \|__\| Pneumology ward  \|__\| Rehabilitation \|__\| Outpatient clinic Was someone accompanying you during the arterial puncture?  \|__\| Yes \|__\| No  If yes:  I__I Senior physician  I__I Resident  I__I Intern  I__I Nurse  I__I Nursing student  Was the patient informed before the arterial puncture?  \|__\| Yes \|__\| No  Was prior anesthesia performed?  \|__\| Yes \|__\| No  If yes, how?  \|__\| Emla patch - \|__\| Emla cream - \|__\| Subcutaneous lidocaine  If yes, how long before?  \|__\| 0 to 30 min - \|__\| 30 to 60 min - \|__\| More than 60 min  Quality of the radial pulse:  I__I Imperceptible, I__I Weak, I__I Normal, I__I Visible  Punctured wrist:  \|__\| Right \|__\| Left  Needle gauge:  \|__\| 25 G: Orange – \|__\| 23 G: Blue – \|__\| Other  Number of attempts before successful arterial puncture:  \|__\| 0 - I__I 1 – I__I 2 – I__I 3  Failure after 3 attempts:  \|__\| Yes \|__\| No If yes, causes:  \|__\| Hematoma \|__\| Insufficient sample, \|__\| Unpuncturable artery \|__\| Artery not found, \|__\| Patient intolerance  What was the course of action after failure?  \|__\| Puncture performed on other wrist  \|__\| Performed with a different needle  \|__\| Performed by another operator  \|__\| Performed at another site (femoral, humeral, capillary)  \|__\| Puncture not performed  **E. Your feelings about this radial arterial puncture during the procedure** Overall fear level (0-10 scale): I__I __I  Fear of causing pain to the patient (0-10 scale): I__I __I  Fear of failing the procedure (0-10 scale): I__I __I  **F. Your feelings about this radial arterial puncture after the procedure**  Satisfaction with your procedure (0-10 scale): I__I __I  Fear of performing the procedure again: (0-10 scale): I__I __I  Confidence in doing the procedure again: (0-10 scale): I__I __I |
| --- | --- |
|  |  |

**Electronic Supplement (ES4). Teaching Slide Deck Used in the Structured Training Program**

Slide presentation used during the structured theoretical training session on arterial puncture. The material was adapted from the New England Journal of Medicine procedural video on radial artery puncture (DOI: 10.1056/NEJMvcm0803851), incorporating annotated screenshots and institutional guidelines. Content was reviewed for accuracy and pedagogical coherence by five academic pulmonologists from Sorbonne Université.


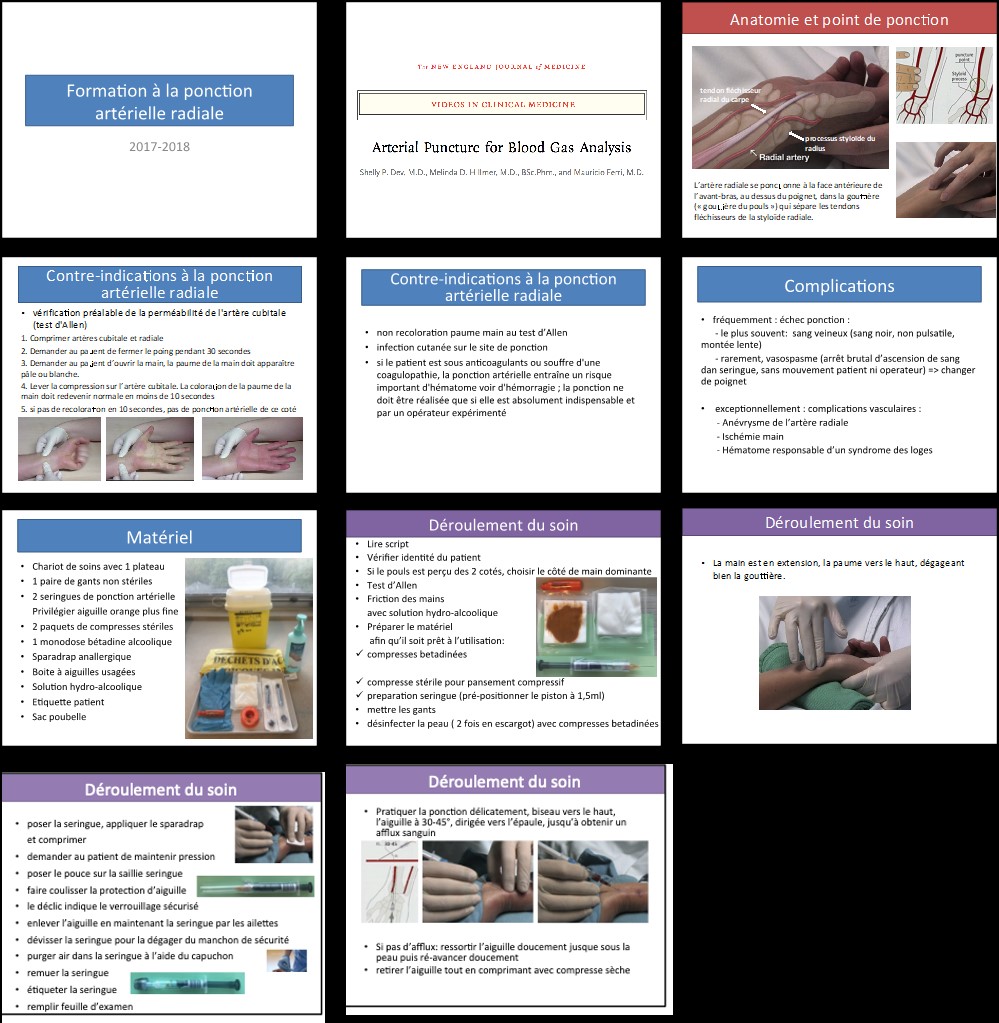


**Electronic Supplement (ES5). Communication Script for Patient Interaction**

Standardized script provided to students during the structured training. The script covers patient introduction, procedural explanation, discussion of potential discomfort, and clarification regarding the absence of validated analgesic strategies. It was written and reviewed for accuracy and pedagogical coherence by the same panel of five academic pulmonologists who evaluated the theoretical training materials. Students first heard the instructor read the script aloud, then practiced it in pairs, and later received a printed copy to review before performing their first arterial puncture on a patient.

***French version (used in the study)***

Bonjour Madame, Bonjour Monsieur.

Comment allez-vous ?....

Je viens vous voir parce que je dois vous faire une prise de sang, pour mesurer les gaz du sang, c'est-à-dire combien il y a d'oxygène et de dioxyde de carbone dans votre sang. C'est une prise de sang différente de celles que l'ont fait d'habitude pour les examens, car le prélèvement se fait dans une artère, au niveau du poignet, et pas dans une veine. C'est un examen important, qui sert à savoir si vos poumons fonctionnent bien, s’ils apportent suffisamment d’oxygène et éliminent bien le gaz carbonique. Cela vas nous aider à comprendre votre essoufflement, à suivre l’évolution de votre maladie, à suivre l’efficacité de vos traitements que vous recevez.

Pour faire ce prélèvement, je vais palper votre pouls au poignet pour bien repérer l'artère. Ensuite, je vais désinfecter, puis faire la piqûre elle-même avec une petite aiguille très fine. L'aiguille passe d'abord la peau, puis entre dans l'artère. Le sang apparait dans la seringue et monte tout seul grâce à la pression qui règne dans l’artère. Vous sentirez l'aiguille passer la peau, comme pour une prise de sang habituelle. Vous pouvez avoir un peu plus mal au moment où elle va rentrer dans l'artère, parce qu'il y a beaucoup de "capteurs de douleur" dans sa paroi. Mais ce n'est pas du tout sûr. Parfois on peut avoir un peu de difficulté à faire entrer l'aiguille dans l'artère si elle est rigide. Il faudra bien que vous me disiez si vous avez mal, et dans ce cas on arrêtera bien sûr. De votre coté, il est important de vous détendre et de respirer calmement, et surtout de ne pas bouger la main pendant que je pique.

Une fois que la seringue sera remplie, je retirerai l'aiguille, et je comprimerai l'endroit de la ponction avec une compresse pendant quelques minutes. Un pansement compressif est ensuite appliqué, pendant une petite heure. Malgré ces précautions, il peut y avoir un hématome après le prélèvement. Ce n'est pas grave et cela disparaît en un ou deux jours.

C'est d'accord pour vous ? Avez-vous des questions ? Surtout n'hésitez pas à me les poser.

Etes vous prêt ? Etes vous bien confortablement installé ? Allez, je vous pique, il n'y en a pas pour longtemps.

Voilà, c'est fait. Je comprime bien. Ça a été ?

***Option*** *: ça a été un peu difficile, je suis désolé. Mais c'est fait, on va avoir le résultat*.

A présent :

Pouvez-vous me dire à combien vous notez votre peur de la ponction artérielle (sur une échelle de 0 à 10; 0 : pas de peur, 10 : peur maximale imaginable) ?

Pouvez-vous me dire à combien vous notez votre douleur lors de la ponction artérielle (sur une échelle de 0 à 10 ; 0 : pas de douleur, 10 : douleur maximale imaginable) ?

A quel moment cette douleur a t’elle été la plus forte :

☐ insertion de l’aiguille ;

☐ pendant ponction ;

☐ retrait de aiguille ;

☐ après ponction ;

☐ lors de répétition de la ponction ;

***English translation (used in the study)***

**Hello Madam, Hello Sir.**

How are you today?

I am coming to see you because I need to draw a blood sample to measure the gases in your blood—that is, the amount of oxygen and carbon dioxide in it. This blood test is different from the usual ones because the sample is taken from an artery in your wrist, not from a vein. It is an important test that helps us determine whether your lungs are working well, whether they bring in enough oxygen and eliminate carbon dioxide properly. It will help us understand your shortness of breath, monitor how your condition is evolving, and assess how effective your treatments are.

To perform this procedure, I will first feel your pulse at the wrist to locate the artery. Then I will disinfect the area and place a very fine needle. The needle first goes through the skin and then into the artery. The blood appears in the syringe and rises on its own because of the pressure inside the artery. You will feel the needle pass through your skin, just like a regular blood draw. You may feel a bit more pain when the needle enters the artery, because its wall contains many “pain receptors.” But that is not always the case. Sometimes it can be a little difficult to enter the artery if it is stiff. It is important that you tell me if you feel pain; if so, we will of course stop.

For your part, it is important to relax, breathe calmly, and especially not move your hand while I am placing the needle.

Once the syringe is full, I will remove the needle and press on the puncture site with a gauze pad for a few minutes. A pressure bandage will then be applied for about an hour. Despite these precautions, a small bruise may appear afterward. This is not serious and usually disappears within one or two days.

Is this acceptable to you? Do you have any questions? Please feel free to ask me anything.

Are you ready? Are you comfortably positioned?

Alright, I am going to proceed with the needle; it will only take a moment.

There you go, it is done. I am applying pressure. How was it?

***Option:*** *It was a bit difficult, I am sorry. But it is done, and we will have the result.*

Now:

Can you tell me, on a scale from 0 to 10 (0 = no fear at all, 10 = the worst fear you can imagine), how much fear you felt about the arterial puncture?

Can you tell me, on a scale from 0 to 10 (0 = no pain at all, 10 = the worst pain you can imagine), how much pain you felt during the arterial puncture?

At what moment was the pain the most intense?

☐ insertion of the needle;

☐ during the puncture;

☐ removal of the needle;

☐ after the puncture;

☐ during repeated puncture attempts.
